# Herpes Simplex Virus-1 ICP27 Nuclear Export Signal Mutants Exhibit Cell Type-Dependent Deficits in Replication and ICP4 Expression

**DOI:** 10.1128/jvi.01957-22

**Published:** 2023-06-13

**Authors:** Leon Sylvester Sanders, Courtney E. Comar, Kalanghad Puthankalam Srinivas, Joseph Lalli, Mark Salnikov, Joy Lengyel, Peter Southern, Ian Mohr, Angus C. Wilson, Stephen A. Rice

**Affiliations:** a Department of Microbiology and Immunology, University of Minnesota Medical School, Minneapolis, Minnesota, USA; b Department of Microbiology, New York University School of Medicine, New York University, New York, New York, USA; University of Virginia

**Keywords:** HSV-1, ICP27, ICP4, molecular genetics, regulation of gene expression

## Abstract

Herpes simplex virus type-1 (HSV-1) protein ICP27 is an essential immediate early (IE) protein that promotes the expression of viral early (E) and late (L) genes via multiple mechanisms. Our understanding of this complex regulatory protein has been greatly enhanced by the characterization of HSV-1 mutants bearing engineered alterations in the ICP27 gene. However, much of this analysis has been performed in interferon-deficient Vero monkey cells. Here, we assessed the replication of a panel of ICP27 mutants in several other cell types. Our analysis shows that mutants lacking ICP27's amino (N)-terminal nuclear export signal (NES) display a striking cell type-dependent growth phenotype, i.e., they grow semi-permissively in Vero and some other cells but are tightly blocked for replication in primary human fibroblasts and multiple human cell lines. This tight growth defect correlates with a failure of these mutants to replicate viral DNA. We also report that HSV-1 NES mutants are deficient in expressing the IE protein ICP4 at early times postinfection. Analysis of viral RNA levels suggests that this phenotype is due, at least in part, to a defect in the export of ICP4 mRNA to the cytoplasm. In combination, our results (i) show that ICP27's NES is critically important for HSV-1 replication in many human cells, and (ii) suggest that ICP27 plays a heretofore unappreciated role in the expression of ICP4.

**IMPORTANCE** HSV-1 IE proteins drive productive HSV-1 replication. The major paradigm of IE gene induction, developed over many years, involves the parallel activation of the five IE genes by the viral tegument protein VP16, which recruits the host RNA polymerase II (RNAP II) to the IE gene promoters. Here, we provide evidence that ICP27 can enhance ICP4 expression early in infection. Because ICP4 is required for transcription of viral E and L genes, this finding may be relevant to understanding how HSV-1 enters and exits the latent state in neurons.

## INTRODUCTION

Herpes simplex virus type-1 (HSV-1) is a widespread human pathogen that infects the majority of the world's population by adulthood (1–3). Primary infection commonly occurs in mucosal epithelia or skin. Following replication at these sites, the virus invades neurons of the peripheral nervous system where it establishes a lifelong latent infection. During latency, viral genomes are harbored in neuronal nuclei as circular episomes, with the expression of the ~85 productive-cycle viral genes being largely repressed. However, periodically, productive-phase gene expression can be reinitiated and viral replication can ensue. Progeny virions can then return to the host periphery to reinfect epithelial cells. Occasionally, the virus targets the eye or central nervous system, where it can cause serious and even life-threatening disease (4–6).

Like other nuclear-replicating dsDNA viruses, HSV-1 uses the host cell RNAP II and associated transcription and mRNA processing machinery to express its genes as mRNAs, as well as host ribosomes for their translation. Viral gene expression occurs in cascade fashion that consists of the ordered expression of IE, E, and L genes. Although the mechanisms that govern the initiation of viral gene expression in latently infected neurons are just beginning to be understood (7, 8), much more is known about how productive replication is induced in epithelial cells and fibroblasts, as this process can be readily studied in tissue culture. In this context, the virion tegument protein VP16 is key to initiating productive infection, as this viral transcription factor drives initial expression of the five IE genes (reviewed in 9, 10). Four of the five IE proteins (ICP0, ICP4, ICP22, and ICP27) then localize to the cell nucleus where they collectively drive the later events of productive infection, including E/L gene expression and viral DNA replication. Perhaps the key IE protein in this regard is ICP4, which serves as the major transcriptional activator protein of HSV-1, being necessary for the transcription of all E and L genes (11, 12). Not surprisingly, ICP4 is essential for productive replication under all known conditions (13).

The only other IE protein which is nonconditionally essential is ICP27 (14–16). It is comprised of 512 amino acid residues and migrates as an ~63 kDa polypeptide on SDS-PAGE. Homologs of ICP27 are conserved in all members of the *Herpesviridae*, the large family of mammalian, avian, and reptilian herpesviruses to which HSV-1 belongs (17, 18). Structurally, ICP27 can be divided into two distinct regions: a disordered N-terminal half (19) and a globular C-terminal half that has been termed the ICP27-homology domain (IHD), as this is the portion of the protein conserved in *Herpesviridae*. Recent work shows that the IHD mediates the dimerization of ICP27, which is critical to its function (17).

ICP27 performs multiple functions during HSV-1 infection, many of which involve posttranscriptional effects on viral and cellular mRNAs (reviewed in 20, 21). At least three properties of the protein are important for these effects. First, ICP27 is an RNA-binding protein that binds to G- or G/C-rich target RNAs via a short RGG-box motif that maps to residues 138 to 152 (22–26). Because the HSV-1 genome has a GC-content of 69%, ICP27 binds avidly to many, if not most viral mRNAs (24, 26). Second, although ICP27's steady-state distribution is predominantly nuclear, it is a nucleocytoplasmic shuttling protein that moves continually between the nucleus and cytoplasm (24, 27, 28). This activity depends on a short N-terminal nuclear export sequence (NES) mapping to residues 4 to 20 (24, 29), although the IHD is also required for shuttling (28). Third, ICP27 binds to numerous host proteins involved in mRNA metabolism (20). These include the C-terminal domain (CTD) of the RNAP II large subunit (30, 31), the poly(A) tail-binding protein (PABP) (32), mRNA export factors (26, 33–35), mRNA splicing proteins (36–38), and components of the mRNA 3′ processing factor cleavage and polyadenylation specificity factor (CPSF) (26).

Perhaps the best characterized posttranscriptional function of ICP27 is its ability to promote viral mRNA export (24). The major mRNA export system in mammalian cells involves a multi-subunit assembly known as the transcription and export complex, or TREX (reviewed in 39, 40). For the export of most cellular mRNAs, TREX components, including the principal RNA adaptor component ALYREF, are recruited to nuclear pre-mRNAs during their biogenesis, with the mRNA splicing step being particularly important for recruitment. The pre-mRNA-TREX complex then binds to the major export receptor NXF1 and its binding partner p15, transferring the bound mRNA to NXF1 for export to the cytoplasm (41). The strong dependence of this system on mRNA splicing is potentially problematic for HSV-1 because most E and L genes lack introns. ICP27 compensates for this by directly binding to viral mRNAs in the nucleus, then recruiting ALYREF through a protein-protein interaction (33). In addition, ICP27 associates with NXF1 itself (42, 43), and this interaction has been shown to be critical for viral mRNA export (34).

Genetic analysis has been key to studying ICP27's many functions. In particular, numerous HSV-1 mutants have been engineered that either fail to express ICP27 or express mutant forms of it (15, 29, 44–47). Although these mutants have been studied in multiple cellular contexts, including HeLa cells and rabbit skin fibroblasts (19, 34, 42, 48), most of the quantitative analysis of mutant replication has been performed in Vero African green monkey kidney cells (15, 29, 44, 45). Although this cell line has been used extensively in HSV-1 research, it is an immortalized line that lacks a functional type I interferon system (49), and thus in many ways does not accurately model natural virus-host interactions. In this study, we sought to characterize the growth of HSV-1 ICP27 mutants in other cell types. In so doing, we discovered that ICP27 NES mutants exhibit a marked cell type-dependent replication phenotype. Surprisingly, these mutants also display a profound defect in ICP4 expression.

## RESULTS

### The HSV-1 ICP27 mutant d1-2 exhibits cell type-dependent growth.

**FIG 1:**
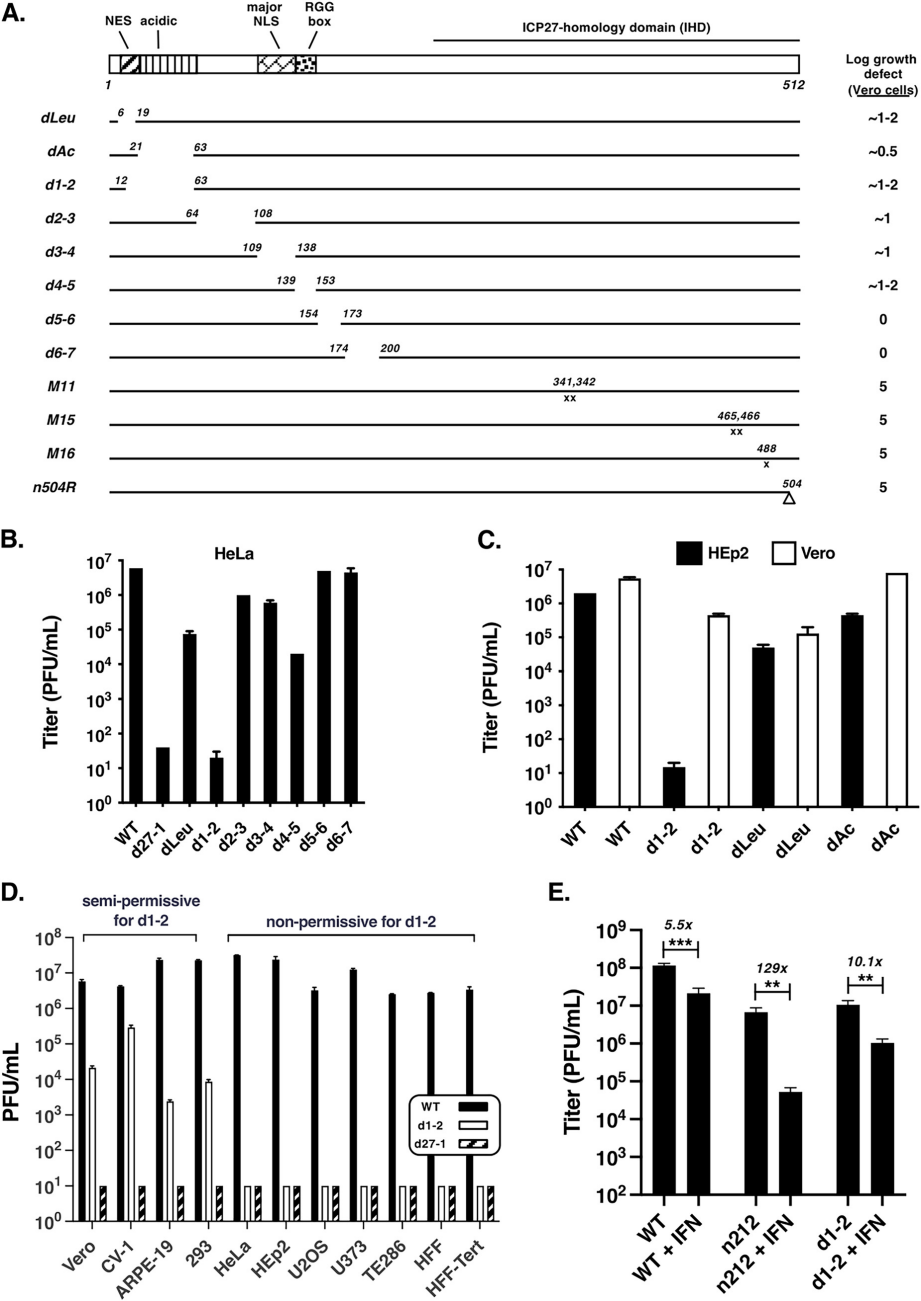
ICP27 mutant d1-2 exhibits cell type-dependent growth. (A) HSV-1 ICP27 mutants used in this study. At the top, a schematic diagram of the 512-residue ICP27 polypeptide is shown, highlighting known domains and functional sequences, including the nuclear export sequence (NES), acidic region, major nuclear localization signal (NLS), RGG-box RNA binding domain, and ICP27-homology domain (IHD). Below are line representations of mutant ICP27 polypeptides, with the numbers indicating the residues deleted (dLeu through d6-7) or mutated in point mutants (M11, M15, M16). In mutant n504R, the number corresponds to the C-terminal residue in the truncated protein, as a result of the insertion of a stop codon linker (inverted triangle). To the right are the mutants’ growth defects (log reductions) in Vero cells, according to previously published studies (15, 29, 45). (B) Growth of mutants in HeLa cells. HeLa cells were infected in biological duplicate at an MOI of 10 and incubated for 24h. The amount of viral progeny in each culture was determined by plaque assay of the harvested cell lysates on ICP27-complementing V27 cells. The bars denote mean titers; error bars denote SEMs. This experiment is representative of two independent experiments that each show d1-2 is unable to replicate to any extent in HeLa cells. (C) Direct comparison of WT and ICP27 mutant growth in HEp2 and Vero cells. Cells were infected in biological duplicate and analyzed as in (A). This experiment was performed one time. (D) Analysis of d1-2 growth in diverse monkey and human cell lines and primary cultures. The indicated cells were infected in biological triplicate at an MOI of 10 PFU/cell, incubated for 1 day, harvested, and titered on V27 cells. Statistical details are as in (A). The limit of detection in these experiments was 10 PFU/mL. This experiment was performed one time for most of the cell lines shown, but was performed twice for HeLa cells and >5 times for HEp2 cells. (E) Analysis of the effects of type-I interferon on d1-2 replication in Vero cells. Triplicate cultures of Vero cells were pretreated with 1,000 U of human α-IFN for 24 h, or mock pretreated, then infected with the viruses shown at an MOI of 10 PFU/cell. At 24 hpi, infections were terminated and replication was assessed by plaque assay of the lysates on appropriate complementing cells. For each viral infection, the fold inhibitory effect of type-I interferon treatment is indicated. The bars indicate mean titers and error bars denote SEMs. Statistical analyses were performed using the Student's *t* test. **, *P* < .01; ***, *P* < .001. This experiment was performed one time.

We previously engineered several HSV-1 ICP27 mutants and characterized their replication phenotypes in Vero cells (15, 29, 44–47). These studies highlighted the functional importance of the IHD, as several point mutations in this sequence (M11, M15, and M16) and even a short C-terminal truncation (n504R) completely abolish HSV-1 growth (5-log reduction, Fig. 1A). In contrast, the N-terminal half of ICP27 is much less sensitive to mutation, with several in-frame deletion mutants in this region being wholly or partially replication-competent in Vero cells. To see if ICP27 N-terminal mutants replicate similarly in a different cellular context, we tested a number of them in HeLa cells, a human tumor line widely used in virology. For comparison, we also included wild-type (WT) HSV-1 (strain KOS1.1, the parental strain of all the mutants used in this study) and the ICP27 null mutant d27-1 (15). The cells were infected at an MOI of 10 PFU/cell and incubated for 24 h, at which time the infections were harvested and titered on the V27 cell line, which is a derivative of Vero cells that complements the growth of ICP27 mutants owing to the expression of a stably integrated ICP27 gene (15). The results (Fig. 1B) showed that, with one exception, all mutants replicate similarly in HeLa cells as they are reported to in Vero cells (Fig. 1A). The exception is d1-2, which has a deletion of ICP27 codons 12 to 63. In Vero cells, this mutant replicates 10- to 100-fold less efficiently than WT HSV-1 (29, 44), but in HeLa cells it replicated ~100,000-fold less efficiently. We next tested the replication of d1-2 in HEp2 cells, another human cancer cell line, and in this case, replication was simultaneously tested in Vero cells (Fig. 1C). As expected, d1-2 replication was modestly deficient in Vero cells. However, its growth in HEp2 cells was highly restricted. In contrast, the replication of dLeu and dAc, which have deletions partially overlapping with that of d1-2, were only modestly impacted by cell type. We considered the possibility that d1-2 had acquired a secondary mutation that reduces its replication in HeLa and HEp2 cells. However, an independent isolate, d1-2b (44), displayed the same growth phenotype (data not shown), strongly arguing against a secondary mutation.

To gain more insight into the cellular properties that influence d1-2's replication, we assessed its growth in several cell lines and primary cell cultures. Each set of cells was infected with d1-2, d27-1, or WT HSV-1, and the infections were incubated for 24 h. The compiled viral yields (Fig. 1D) indicate that WT HSV-1 replicates quite well in all cells tested (>2× 10^6^ PFU/mL), whereas the ICP27 null mutant d27-1 fails to replicate in any context (≤1× 10^1^ PFU/mL). Interestingly, d1-2 fell into two distinct categories with respect to replication. In four cell lines (Vero, CV-1, ARPE-19, and 293), it replicates semipermissively, yielding >2× 10^3^ PFU/mL. However, in five other cell lines (HeLa, HEp2, U2OS, U373, HFF-Tert), and two primary human cell cultures (TE286 tonsillar fibroblasts and HFF), it fails to replicate to any extent, yielding <1× 10^1^ PFU/mL. These results confirm that d1-2 exhibits a cell-type dependent replication phenotype and identify several human cell lines and primary cells that are completely nonpermissive for this mutant.

An unusual property of Vero cells is that they are β-interferon gene deletion mutants, and hence, are unable to synthesize type-I interferon in response to viral infection (49). However, these cells are capable of mounting an antiviral response when treated with exogenous type-I interferon (50, 51). To investigate whether the interferon deficiency of Vero cells explains their ability to serve as semipermissive hosts for d1-2, we asked if pretreating these cells with type-I interferon would restrict d1-2 growth. For comparison, we included two other viruses in our analysis: WT HSV-1, which is relatively resistant to type-I interferon (50–52), and n212, an HSV-1 ICP0 mutant that is hypersensitive to type-I interferon (50). Thus, Vero cells were pretreated or not with type-I interferon for 24 h, then infected with WT HSV-1, n212, or d1-2. The infections were harvested at 24 hpi and viral replication was determined by plaque assay (Fig. 1E). The results showed that interferon pretreatment led to a modest 5.5-fold reduction in WT HSV-1 replication and a much larger 129-fold reduction for n212. Type-I interferon pretreatment reduced d1-2 replication by 10.1-fold, similar to what was seen for the WT virus. We conclude that d1-2 can still replicate to an appreciable extent in Vero cells even when the type-I interferon system has been activated prior to infection.

### The ICP27 cell-dependent growth phenotype maps to the NES.

**FIG 2:**
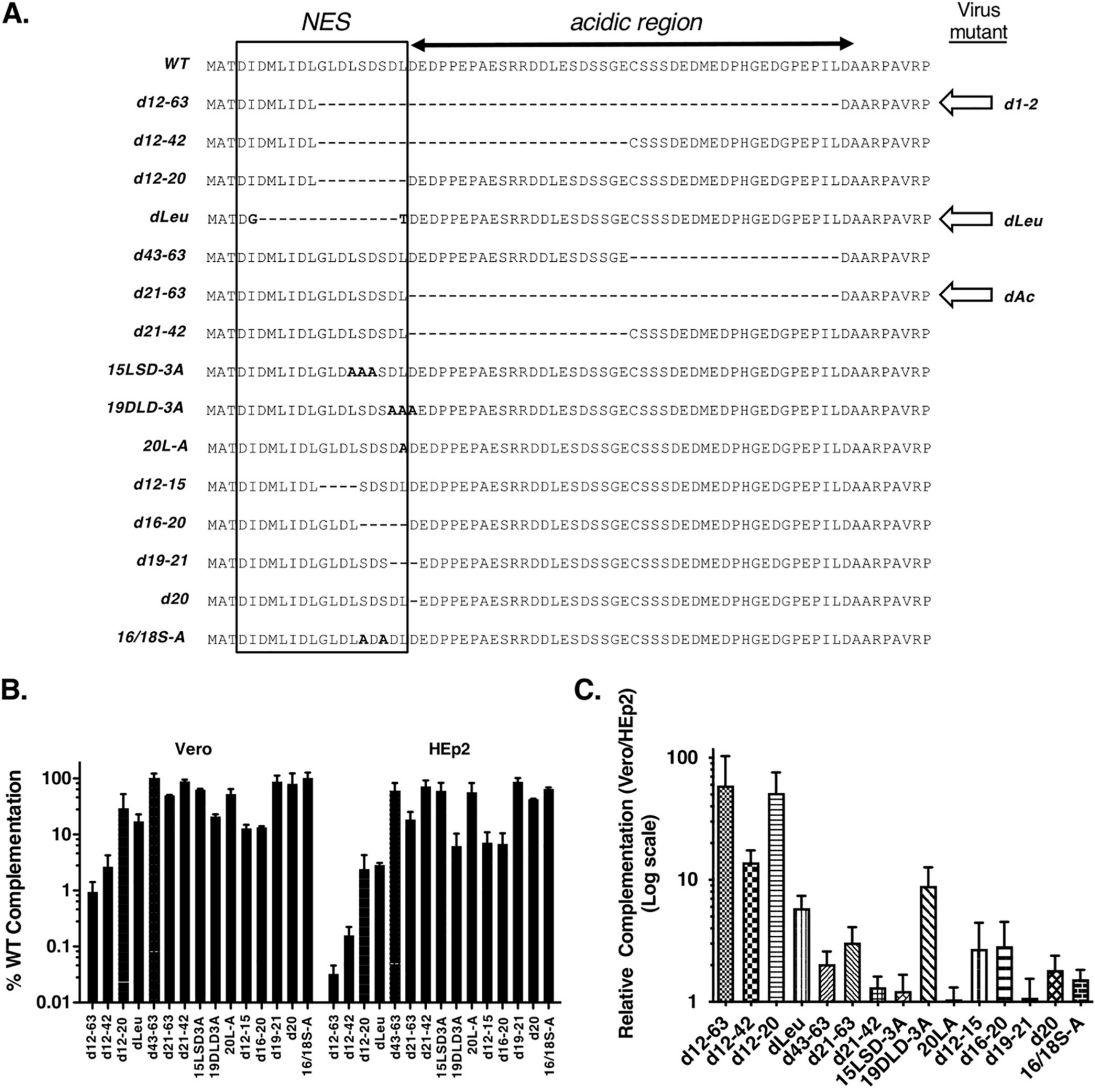
The ICP27 sequences associated with cell-type dependent replication map to the NES. (A) Mutations in ICP27 expression plasmids used in the analysis. The diagram shows the sequences at the N termini of WT ICP27 or various mutant proteins; dashes denote deletions and bold letters denote amino acid mutations. The ICP27 NES and acidic region are indicated. At the right, the block arrows denote HSV-1 ICP27 mutants that possess the indicated sequence alterations. (B) Viral complementation analyses. HEp-2 and Vero cells were transfected in biological triplicate with WT or ICP27 mutant plasmids. After 1 day, cells were infected with d27-1 and incubated for 24 h before being harvested. Cell lysates were titered on V27 cells to determine viral growth, and the mean titers of the mutant plasmid transfection samples were divided by the mean titer of the WT ICP27 plasmid transfection samples (and multiplied by 100 to express as a percentage). The values shown represent the means of two to four independent experiments for each plasmid, and error bars denote SEMs. (C) Relative complementation in Vero versus HEp2 cells. To determine this, the relative complementation of each plasmid in Vero versus Hep2 cells was determined for each of the two to four independent experiments. The means were then determined; error bars show SEMs.

To better define the N-terminal ICP27 sequences which determine cell type-dependent growth, we took advantage of the fact that transfection of an ICP27 expression plasmid can complement the growth of an HSV-1 ICP27 null mutant (44, 53). Thus, several N-terminal deletion or substitution mutations were engineered into an ICP27 expression plasmid (Fig. 2A). One of the plasmids, pd12-63, exactly recreated the deletion in d1-2 (see arrow in Fig. 2A). Additionally, we used a previously isolated ICP27 expression plasmid encoding the dLeu form of ICP27, which is deleted for residues 6 to 19 and carries point mutations at residues 5 and 20 due to the introduction of a restriction enzyme site (29). To carry out the complementation studies, the mutant plasmids were transfected into both Vero and HEp2 cells, as models of semipermissive and restrictive cells, respectively. As controls, additional cultures were transfected with a WT ICP27 expression plasmid or vector-only control. One day later, the transfected cells were infected with d27-1 and incubated for an additional day before being harvested to determine the viral titers on V27 cells. The compiled results, expressed as percent complementation compared to the WT plasmid are shown in Fig. 2B (note that the scale is in log-10 form). We first analyzed the data to identify the N-terminal sequences which are important for replication in each cell line. The conclusion was the same for both cell types: the NES is the functionally important sequence. This is supported by the fact that the plasmids which exhibited the poorest complementation all had NES mutations (d12-63, d12-42, d12-20, dLeu, d12-15, d16-20, and 19DLD3A). In contrast, mutations that only affected ICP27's acidic region (d43-63, d21-63, and d21-42) had negligible or only modest effects on complementation in both cell lines.

The data were next analyzed to determine which mutations are most strongly associated with cell type-dependent growth. To do this, we calculated the relative complementation of each plasmid in Vero versus HEp2 cells (Fig. 2C). The five mutations which exhibited the strongest cell-type dependence all deleted or altered the NES (d12-63, d12-20, d12-42, 19DLD-3A, and dLeu, which exhibited Vero/HEp2 complementation ratios of 59-, 52-, 14-, 8.9-, and 5.9-fold, respectively). These results indicate that the cell-type dependent replication is associated with mutation of the NES.

One aspect of this analysis that deserves comment is the relatively modest 5.9-fold effect of the dLeu mutation on cell type-dependence (Fig. 2C). This is surprising because the d12-20 mutation, which has a similar but smaller NES deletion, exhibits a much more striking 52-fold effect. One possible explanation relates to the leucine (L)- and aspartic acid (D)-rich nature of the NES, and the fact that deletions, by their nature, juxtapose polypeptide sequences that are normally separated. In the case of dLeu, the 14-residue deletion gives rise to an ICP27 molecule with the N-terminal sequence MATDGTDEDPPEPAESRRDDL. This has the residues DDL, which are normally found at positions 34 to 36, repositioned at residues 19 to 21. Our analysis indicates that, in the WT protein, NES residues 19 to 21 (DLD) are functionally quite important, because changing these to alanine (A) in mutant 19DLD3A results in markedly reduced complementation (Fig. 2B). This suggests that the unexpectedly modest cell-type dependence of dLeu could be a fortuitous effect of replacing the critical DLD sequence with the related sequence DDL.

### The d1-2 mutant is tightly blocked for viral DNA replication in HEp2 but not Vero cells.

**FIG 3:**
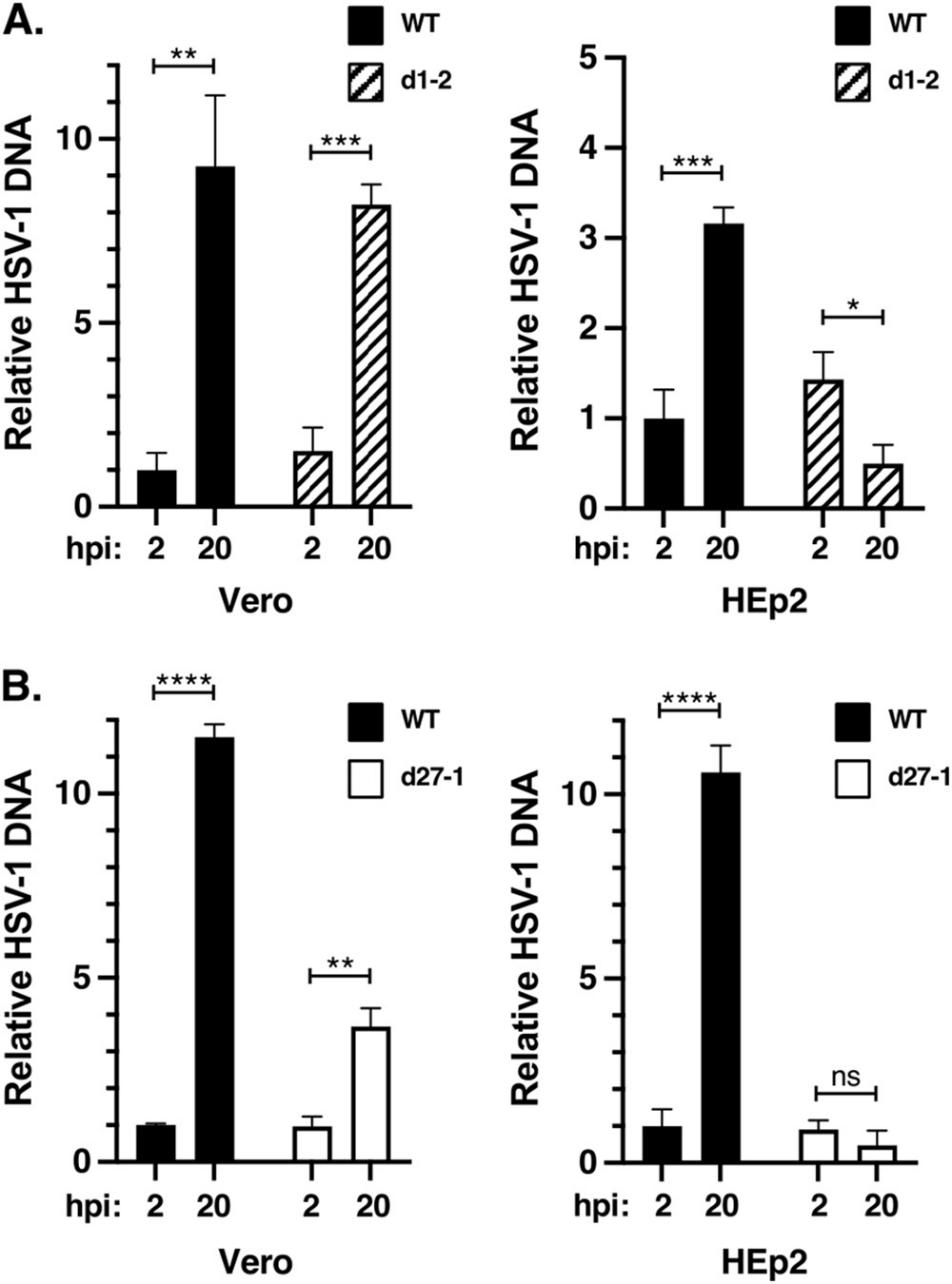
ICP27 mutants d1-2 and d27-1 can replicate their DNA in Vero but not HEp2 cells. Vero or HEp2 were infected in biological triplicate at an MOI of 10 PFU/cell with WT HSV-1 and ICP27 mutant d1-2 (A) or d27-1 (B). Total infected cell DNA was isolated at 2 and 20 hpi and the HSV-1-specific DNA at each time point was quantitated by qPCR. The data were normalized to a single-copy cellular gene to correct for differences in cell recovery. Vero and HEp2 data were generated in different qPCR runs, so the results are shown in separate graphs. For each analysis, the level of WT HSV-1 DNA at 2 hpi was assigned the value of 1.0. The bars denote means, and error bars show SEMs. Statistical analyses were performed using the Student's *t* test. *, *P* < .05; **, *P* < .01; ***, *P* < .001; ****, *P* < .0001; ns (not significant), *P* > 0.05. This experiment is representative of two independent experiments that each show that d1-2 and d27-1 are able to replicate their DNA to some extent in Vero cells but not at all in HEp2 cells.

The above results demonstrate that the d1-2 mutant is severely restricted for growth in HEp2 and several other human cells, but replicates semipermissively in Vero and some other cell lines. To understand the molecular basis of this phenotype, we sought to compare d1-2 infections in restrictive HEp2 cells to those in semipermissive Vero cells. We first tested viral DNA replication, as this is a central event in the HSV-1 replication cycle. In addition, prior work suggests that ICP27 mutants differ in their ability to carry out DNA replication in these two cell lines. In Vero cells, viral DNA replication is known to proceed in the absence of ICP27, but at a reduced rate compared to the WT infection (15, 16). Consistent with this, we found that both d27-1 and d1-2 are capable of some genome amplification in Vero cells (29, 44). In contrast, we reported that in HEp2 cells, little if any viral DNA is replicated by either mutant (54). However, our previous studies did not include a side-by-side comparison of ICP27 mutant DNA replication in the two cell lines. To carry out such an analysis for d1-2, we infected replicate cultures of Vero or HEp2 cells with WT HSV-1 or d1-2 and isolated total cellular DNA at 2 hpi (input DNA) and 20 hpi (input + replicated DNA). The relative level of HSV-1 DNA in these preparations (normalized to a single-copy host cell gene) was quantitated by qPCR, with the 2 hpi level of viral DNA in the WT infection being assigned the value of 1.0 (Fig. 3A). The results agree with previous findings, showing that WT HSV-1 is capable of replicating its DNA in both cell lines, but that d1-2 is only able to amplify its genome in Vero cells. In fact, the amount of HSV-1 DNA in d1-2-infected HEp2 cells decreased 2.9-fold over the course of infection, suggesting degradation of some of the input DNA. These results provide an explanation for the failure of d1-2 to replicate to any extent in HEp2 cells. We next repeated the analysis for the ICP27 null mutant d27-1 (Fig. 3B). Again, the results were consistent with previous analyses, showing that d27-1 is capable of significant DNA replication in Vero but not HEp2 cells.

### HSV-1 ICP27 NES mutants are deficient in the early expression of ICP4.

**FIG 4:**
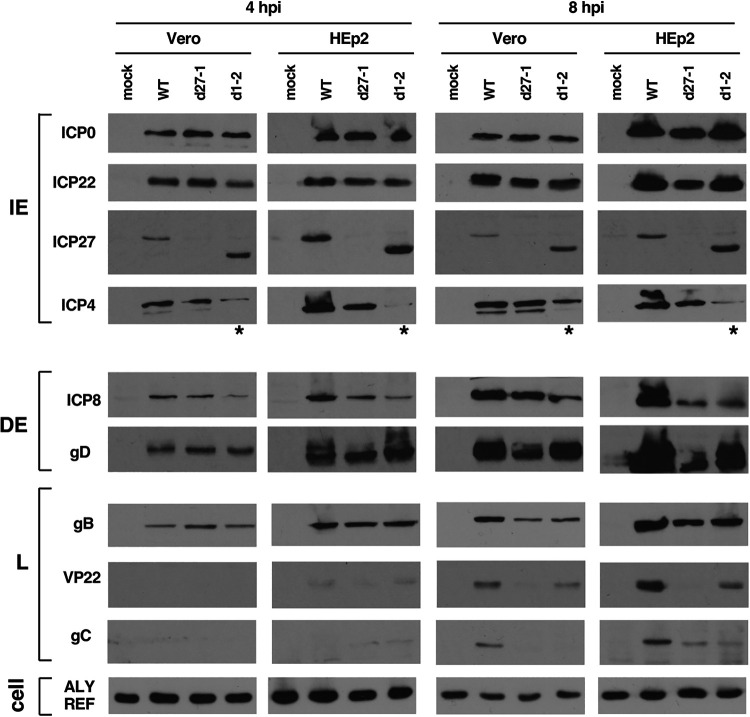
ICP4 expression is deficient in d1-2-infected cells. Vero or HEp2 cells were infected at an MOI of 10 PFU/cell as indicated and total proteins were harvested at 4 and 8 hpi. Protein accumulation was assessed by immunoblotting. Lanes showing abnormally low expression of ICP4 are indicated with an asterisk. Data shown are from a single infection, but the ICP4 results are representative of those obtained in numerous (>8) similar immunoblotting experiments.

The failure of d1-2 to replicate its DNA in HEp2 cells could be due to an upstream block in viral gene expression. To investigate this, we used immunoblotting to characterize HSV-1 protein expression in d1-2-infections. Cultures of Vero or HEp2 cells were mock infected or infected with d1-2, d27-1, or WT HSV-1, and total proteins were isolated at 4 and 8 hpi. We first analyzed IE proteins (Fig. 4, top panels). In both Vero and HEp2 cell lines, d1-2 showed robust expression of ICP22 and ICP0. Likewise, ICP27 was expressed efficiently in d1-2-infected cells (note that the ICP27 molecule encoded by d1-2 migrates more rapidly than the WT protein). The results for ICP4, however, were quite different. In HEp2 cells, ICP4 expression was reduced in d1-2-infected cells at both 4 and 8 hpi (Fig. 4, denoted by asterisks). In Vero cells, ICP4 expression was also reduced in the d1-2 infections (Fig. 4, asterisks), but the effect was not as dramatic as in HEp2 cells, especially at 8 hpi. We also examined DE proteins ICP8 and gD, and L proteins gB, VP22, and gC (Fig. 4, middle and bottom panels). The levels of these viral polypeptides, with the exception of gD and gB, were reduced to varying extents in the ICP27 mutant-infected cells, consistent with previous analyses (29, 44).

**FIG 5:**
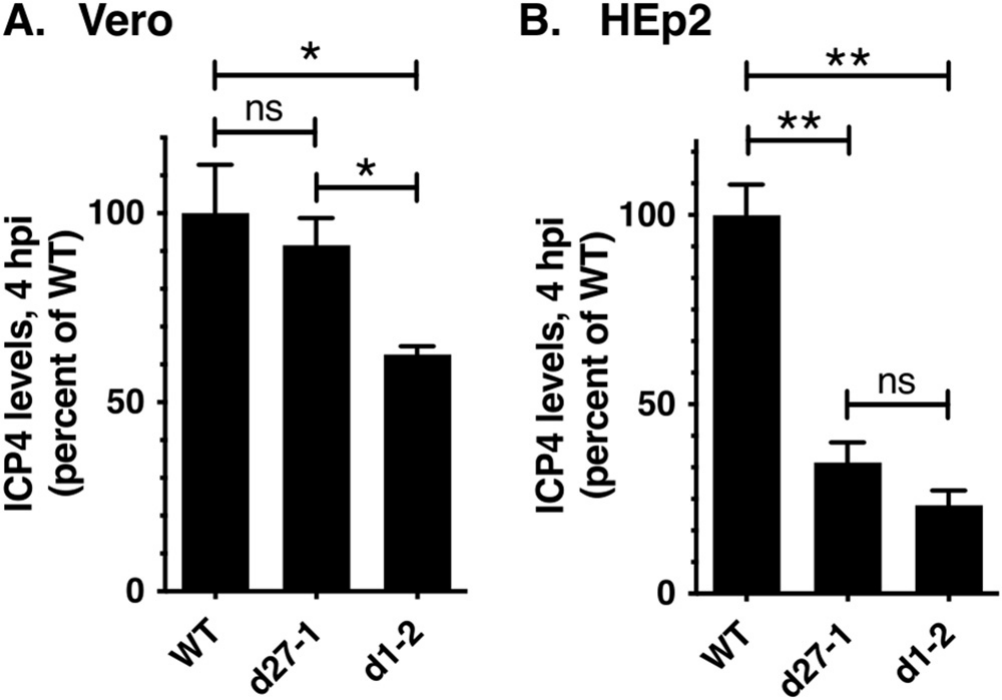
Quantitative analysis of ICP4 expression in ICP27 mutant infections. HEp2 or Vero cells were infected in biological triplicate as shown, and total protein extracts were prepared at 4 hpi. ICP4 and ALYREF levels were determined by immunoblotting; the resulting data were analyzed using ImageJ software. To correct for differences in cell recovery, ICP4 signals were normalized to those of the ALYREF loading control on the same filter. The bars denote means, and error bars show SEMs. Statistical analyses were performed using the Student's *t* test. *, *P* < 0.05; **, *P* < 0.01; ns (not significant), *P* > 0.05. This comparative quantitative analysis of ICP4 expression was performed one time.

**FIG 6:**
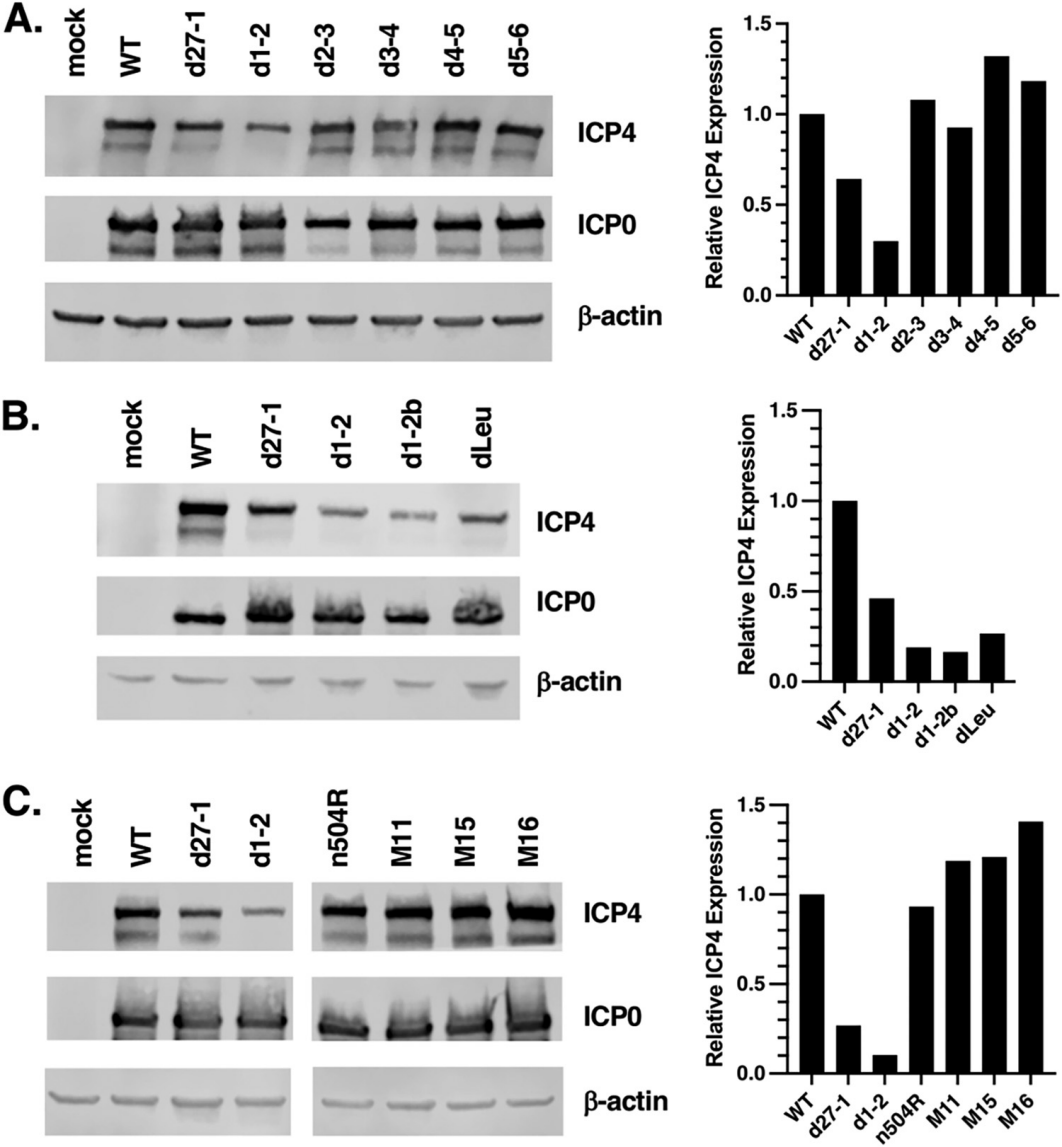
ICP4 expression is deficient in a subset of HSV-1 ICP27 mutants. Single cultures of HEp2 cells were mock infected or infected with the viruses indicated. Total proteins were isolated at 4 hpi and analyzed by immunoblotting for IE proteins ICP4 and ICP0, and β-actin as a loading control (left part of figure). Relative ICP4 levels were quantitated by ImageJ analysis (right part of figure); data were corrected for cell recovery by normalizing to β-actin signals. (A) Analysis of ICP27 N-terminal mutants; (B) Analysis of ICP27 NES mutants; (C) Analysis of ICP27 C-terminal mutants. Each of these experiments was performed one time; however, three independent experiments confirmed that N-terminal mutant dLeu is deficient in ICP4 expression at 4 hpi in HEp2 cells.

The poor expression of ICP4 in d1-2-infected cells at early times postinfection was consistently observed in several similar immunoblotting experiments (data not shown). These results were surprising because, to our knowledge, ICP27 has not been previously implicated in enhancing ICP4 expression. To pursue this further, we carried out a quantitative immunoblotting analysis. Triplicate cultures of Vero or HEp2 cells were infected with WT HSV-1, d27-1, or d1-2, and total proteins were harvested at 4 hpi. The samples were analyzed by immunoblotting and ICP4 bands were quantitated by image analysis. The results differed between the two cell lines (Fig. 5). In Vero cells, ICP4 levels were significantly reduced only in the d1-2 infection, to 63% of the WT level. These data suggest that the d1-2 ICP27 polypeptide actively interferes with early ICP4 expression in Vero cells. However, in HEp2 cells, ICP4 expression was found to be deficient in both the d27-1 and d1-2 infections, to 35% and 23%, respectively, of WT levels. The deficient expression of ICP4 in d27-1-infected HEp2 cells at 4 hpi was consistently observed in multiple repeat experiments (e.g., this defect can be noted in Fig. 4 and is documented in Fig. 6). However, the extent of the deficiency was somewhat variable between experiments, perhaps due to differences in monolayer confluence or cell passage number. The finding that ICP4 levels are reduced in d27-1 infected HEp2 cells indicates that, in these cells, the early expression of ICP4 is dependent on ICP27 and that this function is lacking in the d1-2 mutant.

We next surveyed our collection of HSV-1 ICP27 mutants to see if any others exhibit deficient expression of ICP4 in HEp2 cells. In the first experiment, mutants in the N-terminal half of ICP27 (d1-2, d2-3, d3-4, d4-5, d5-6) were examined. Following infection, proteins were collected at 4 hpi and ICP4, ICP0, and β-actin levels were assessed by immunoblotting (Fig. 6A, left). ICP4 bands were quantitated by image analysis, using β-actin levels to correct for differences in cell recovery (Fig. 6A, right). Although the d27-1 and d1-2 mutants again showed deficiencies in ICP4 accumulation, none of the other mutants exhibited this phenotype. We next tested ICP27 mutants having alterations in the NES region (Fig. 6B). Both d1-2 and its independent isolate d1-2b showed deficient ICP4 expression, as did the NES mutant dLeu. Lastly, we analyzed ICP27 mutants with alterations in the C-terminal half of ICP27 (M11, M15, M16, and n504R) (Fig. 6C). All expressed ICP4 robustly at 4 hpi, whereas d27-1 and d1-2 were again deficient (Fig. 6C). Together, these results show that only a subset of ICP27 mutants exhibit deficient ICP4 expression in HEp2 cells. This phenotype is associated with mutants that lack ICP27 entirely (d27-1) or that have NES mutations (d1-2 and dLeu).

### Complementation of the d1-2 ICP4 expression defect fails to rescue virus growth.

**FIG 7:**
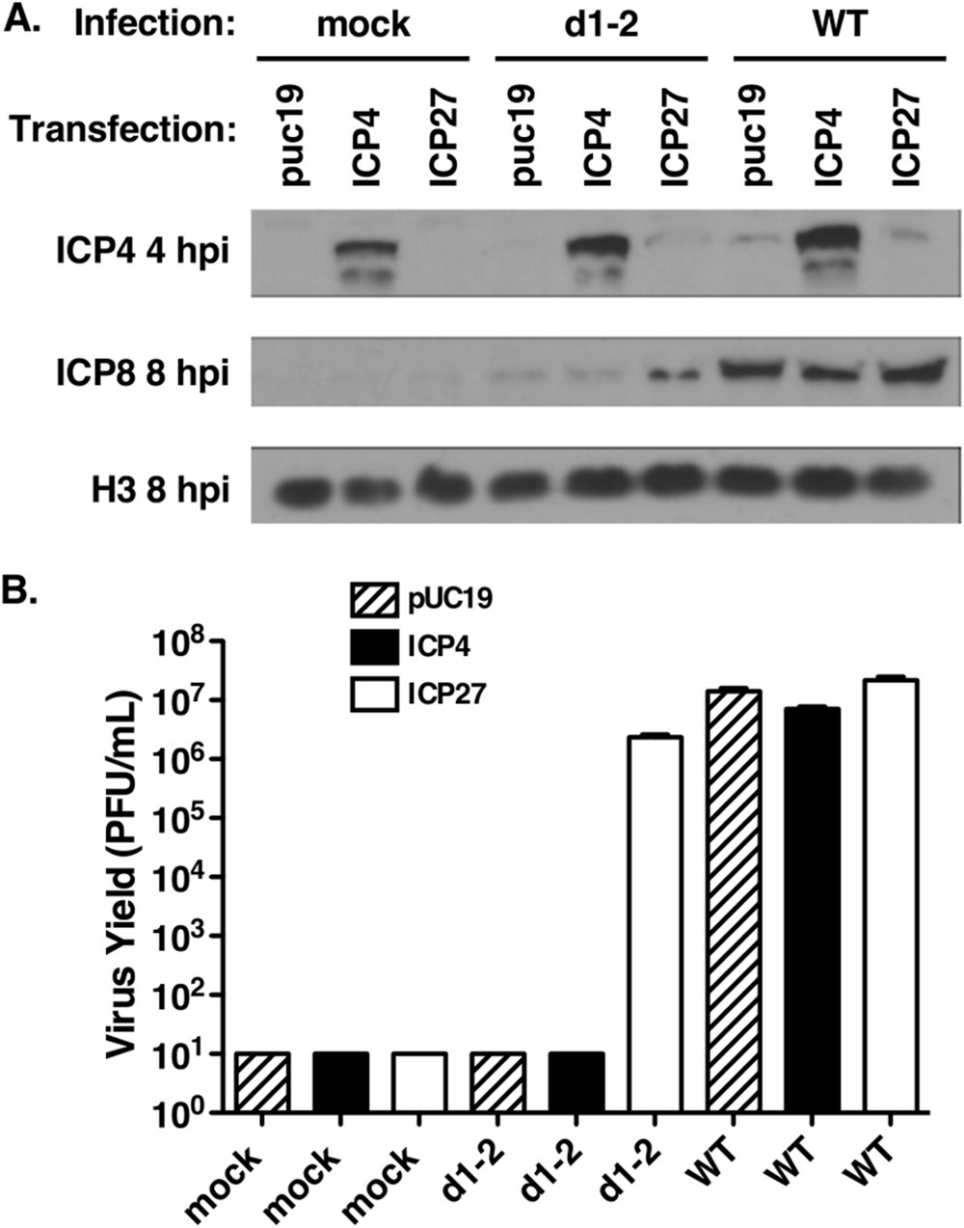
Ectopic expression of ICP4 fails to complement d1-2 in HEp2 cells. Hep2 cells were transfected in quintuplicate with ICP4 or ICP27 expression plasmids, or with pUC19 as a negative control. One day later, the cells were mock infected or infected with d1-2 or WT HSV-1 at an MOI of 10. At both 4 and 8 hpi, one of the replicate cultures were prepared for protein analysis, and the remaining three replicates were harvested at 24 hpi and subjected to plaque assay on V27 cells. (A) Immunoblot analysis. (B) Titer of infections. The expression plasmids transfected in each culture are indicated. The means of triplicate infections are shown; error bars denote SEMs. The limit of detection in the plaque assays was 10 PFU/mL. This experiment was performed one time.

Based on the above results, it is conceivable that the inability of d1-2 to replicate in HEp2 cells is due to its deficient expression of ICP4, which is a key inducer of E/L gene expression, and hence, a required factor for viral DNA replication. To test this hypothesis, we carried out a plasmid complementation experiment. Replicate cultures of HEp2 cells were transfected with pUC19 as a negative control, or with expression plasmids for ICP4 or ICP27. One day later, the cells were mock infected or infected with d1-2 or WT HSV-1. To assay protein expression, some cultures were harvested at 4 or 8 hpi for immunoblot analysis (Fig. 7A), while the rest were harvested at 24 hpi to assay viral growth (Fig. 7B). Although transfection of the ICP27-expressing plasmid resulted in near complete restoration of d1-2 growth, transfection of the ICP4 plasmid had no enhancing effect. This was not due to insufficient ICP4 expression, as ICP4 levels were significantly elevated in all cultures receiving the ICP4 plasmid (Fig. 7A). Interestingly, in d1-2-infected cells, the low ICP8 expression at 8 hpi was not rescued by ectopic expression of ICP4, suggesting that its expression defect is not due to insufficient ICP4. These results demonstrate that the replication defect of d1-2 in HEp2 cells is not solely due to poor ICP4 expression.

### The d1-2 mutant is trans-dominant.

**FIG 8:**
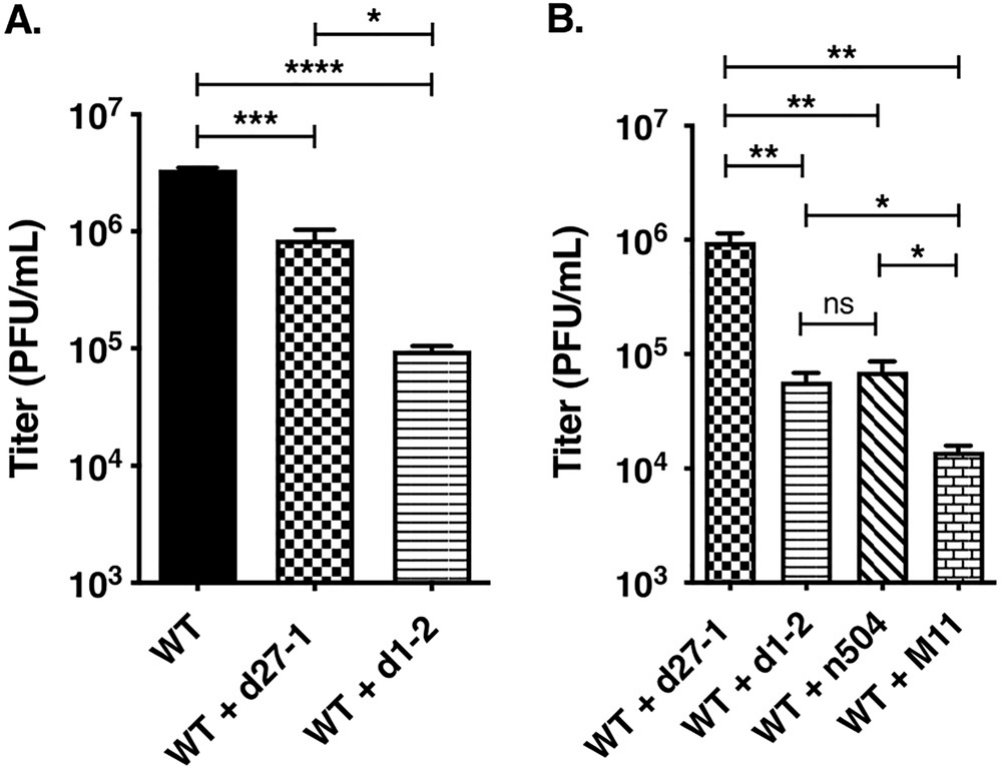
The d1-2 mutant is trans-dominant. (A) Co-infection assay to assess the effects of d27-1 and d1-2 on WT HSV-1 infection. Triplicate cultures of HEp2 cells were infected with WT HSV-1 at an MOI of 1 PFU/cell, minus or plus co-infection with d27-1 or d1-2 at an MOI of 10 PFU/cell. At 24 hpi, infections were harvested and cell lysates were subjected to plaque assay on Vero cells to assess the replication of WT HSV-1. Note that d1-2 produces minute plaques under these conditions (29) which were not scored. (B) Co-infection assay to assess the trans-dominance of d1-2, n504R, and M11. The experiment was carried out as in (A), except that the WT HSV-1 infection alone was omitted. For both (A) and (B), the means of triplicate infections are shown; error bars show SEMs. Statistical analyses were performed using the Student's *t* test. *, *P* < .05; **, *P* < .01; ***, *P* < .001; ***, *P* < .0001; ns (not significant), *P* > 0.05. This experiment was performed one time.

Our analysis of ICP4 expression in d1-2 infections suggests that its altered ICP27 polypeptide can actively interfere with ICP4 expression. This is turn raises the possibility that the d1-2 protein is trans-dominant, i.e., capable of inhibiting the function of the WT protein and/or the replication of WT HSV-1. There is precedence for trans-dominant mutations in ICP27, as Smith et al. showed that ICP27 genes bearing short amino acid insertions in residues 260 to 434 can interfere with ICP27 regulatory activities and inhibit HSV-1 replication (55). To test whether d1-2 exhibits trans-dominance, we carried out a co-infection assay wherein HEp2 cells were co-infected with WT HSV-1 and various replication-defective ICP27 mutants, including d1-2. To increase the opportunity to observe inhibition of WT HSV-1 growth, the WT virus was infected an MOI of 1 PFU/cell and the mutants were infected at an MOI of 10 PFU/cell. In the first experiment, the potential inhibitory effects of d27-1 and d1-2 were studied (Fig. 8A). Although both mutants interfered with WT growth, d1-2 was significantly more repressive, showing a 35-fold reduction compared to a 4.0-fold reduction by d27-1. As d27-1 is unable to produce an ICP27-related protein, this difference can be attributed to the trans-dominance of the d1-2 ICP27 polypeptide. Next, we compared the trans-dominance of d1-2 to those of two other nonreplicating ICP27 mutants, n504R and M11, both of which have alterations in the C-terminal half of ICP27 (Fig. 1A). All three mutants inhibited WT HSV-1 infection significantly compared to null mutant d27-1 (Fig. 8B). The highest degree of trans-dominance was exhibited by M11, which has two amino acid alterations at residues 341/342. Interestingly, this is in the same region of ICP27 that was identified by Smith et al. as being the site of trans-dominant mutations (55). Together, these experiments show that the d1-2 mutant is trans-dominant. However, this property is not unique to d1-2 but is shared with some other ICP27 mutants, one of which (M11) exhibits a more extreme phenotype.

### Evidence that ICP4 mRNA export is defective in d1-2-infected HEp2 cells.

**FIG 9:**
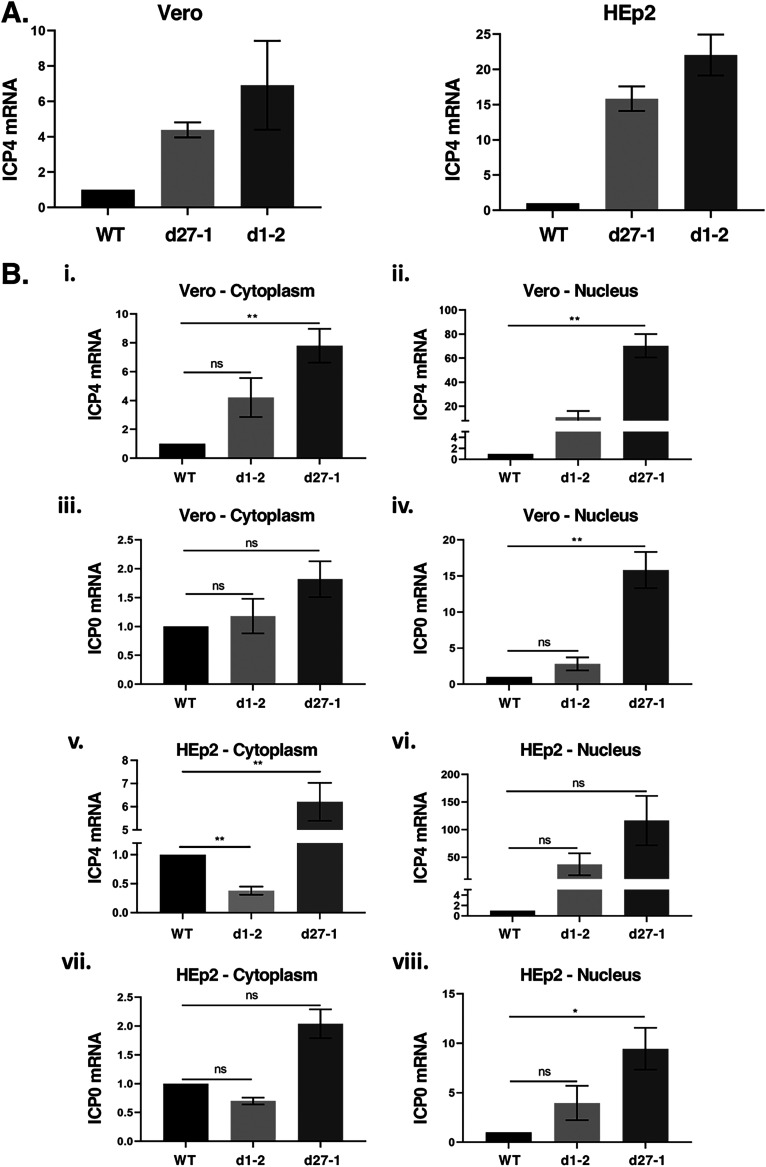
ICP4 mRNA expression in ICP27 mutant infections. Vero or HEp2 cells were infected with the viruses indicated at an MOI of 10 PFU/cell, and infected cell mRNA was isolated at 3 hpi and quantitated by RT-qPCR as described in *Materials and Methods*. Three identical independent experiments were performed; the data shown are derived from these experiments. (A) Analysis of total cellular RNA. (B) Analysis of cytoplasmic and nuclear RNA fractions. For both (A) and (B), viral mRNA levels were normalized to 18S rRNA levels in the same samples, and are expressed as fold change compared to the WT HSV-1 infection, which was assigned the value of 1.0.

Our results show that ICP4 expression can be decreased by certain mutations in ICP27. To gain insight into the mechanisms involved, we assessed ICP4 mRNA accumulation in ICP27-mutant infected cells. In the first set of experiments, both Vero and HEp2 cells were infected with WT HSV-1, d27-1, or d1-2, and total cellular RNA was harvested at 3 hpi. This time point was chosen because mRNA expression precedes protein expression, and we consistently observed a significant ICP4 protein defect at 4 hpi. Following purification of the RNA, ICP4 levels were quantitated by RT-qPCR (Fig. 9A). The results showed that total ICP4 mRNA levels are significantly elevated in the mutant-infected cells compared to the WT-infected cells (note that the level of ICP4 mRNA in WT HSV-1 infected cells was assigned the value of 1.0). Overexpression of ICP4 mRNA by the mutants was seen in both cell lines, although the effect was more pronounced in HEp2 infections (15- to 20-fold overexpression compared to 5- to 7-fold in Vero). These results were unexpected given that ICP4 protein expression is reduced at 4 hpi in d1-2-infected Vero cells and in d27-1/d1-2-infected HEp2 cells (Fig. 5). On the other hand, these findings are consistent with prior analyses showing that ICP27 negatively regulates IE gene expression during the E and L phases of viral infection (14, 15).

ICP27 is capable of enhancing the export of viral mRNAs to the cytoplasm (24, 56, 57), as well as increasing their translation once they have reached this compartment (24, 56, 57). Because either or both of these activities could influence ICP4 expression, it is critical to ascertain the cellular localization of the ICP4 mRNA produced in the mutant infections. Therefore, we carried out a second set of analyses in which we fractionated the infected cell material into soluble (cytoplasmic) and particulate (nuclear) fractions prior to RNA purification. The fractionated RNA was then analyzed by RT-qPCR to measure ICP4 mRNA levels. ICP0 mRNA levels were also determined as a control. Fig. 9B presents the results of these analyses in Vero (panels i-iv) and HEp2 (panels v-viii) cells. Note that for each analysis, the levels of IE mRNA are normalized to 18S rRNA levels in the same sample, and for this reason, the RNA levels in nuclear and cytoplasmic fractions cannot be directly compared. In Vero cells, d1-2 showed higher levels of cytoplasmic ICP4 mRNA relative to the WT virus but this increase was not statistically significant (panel i), despite expressing lower levels of ICP4 protein at 4 hpi (Fig. 4 and 5). This suggests that ICP4 mRNA is not translated efficiently in d1-2-infected Vero cells. It is also notable that there is significantly more (8-fold) ICP4 mRNA in the cytoplasm of d27-1-infected cells than in the cytoplasm of WT-infected cells (Fig. 9, panel i), but that ICP4 protein is not overexpressed by this mutant. This could also be explained by a reduced rate of ICP4 mRNA translation in the absence of ICP27.

In HEp2 infections, the results were somewhat different. The level of cytoplasmic ICP4 mRNA in the d1-2 infection was significantly depressed compared to the WT infection, being 38% of the WT level (Fig. 9B, panel v). This reduction is consistent with the diminution of ICP4 protein level in d1-2 infections at 4 hpi, which was 23% of the WT level (Fig. 5). Of note, the reduction in cytoplasmic ICP4 mRNA seen in the d1-2 infection was not observed in the nuclear fraction (panel vi), and was specific for the ICP4 transcript, as cytoplasmic ICP0 mRNA levels were similar to those in the WT infection (panel vii). It should be also noted that the reduction in cytoplasmic ICP4 mRNA was not seen in d27-1-infected cells, which showed elevated cytoplasmic ICP4 mRNA compared to the WT infection (panel v). Together, these results demonstrate that ICP4 mRNA levels in soluble, cytoplasmic fractions are reduced in d1-2-infected HEp2 cells compared to WT virus-infected cells, despite overall higher levels of total ICP4 mRNA. This is consistent with a defect in ICP4 mRNA export.

## DISCUSSION

### The ICP27 NES is essential for HSV-1 replication in many human cells.

The ability of a virus to replicate in a specific animal species or its cells is known as the viral “host-range” (58). Mutations in viruses which alter host-range are of interest as their study can help elucidate cellular pathways impacting viral infection. Here, we evaluated the growth of a panel of HSV-1 ICP27 mutants, previously characterized in monkey-derived Vero cells, in multiple cellular contexts. Remarkably, while the ICP27 d1-2 mutant grew at a low, but measurable level in Vero cells, it was completely blocked from replicating in most human cells tested, including primary fibroblasts. Further work showed that this host-range phenotype maps to ICP27's N-terminal NES. These findings expand and redefine our understanding of ICP27's functional domains by showing that its NES is indispensable for ICP27 function in many, if not most, human cells. The NES is thought to mediate ICP27’s nuclear export activity through a physical interaction with NXF1 (42, 59). This host cell protein, together with its heterodimeric partner p15, are vital components of the TREX mRNA export machinery and constitute the major transporter of spliced cellular mRNAs in mammalian cells (24, 29, 33, 34). ICP27’s interaction with NXF1 is proposed to allow unspliced HSV-1 E/L transcripts access to this same pathway (42, 59). Consistent with such a critical function, the NES is highly conserved in ICP27 homologs of the Simplexviruses, the herpesvirus genus to which HSV-1 belongs. Multiple alignment analysis reveals a core motif of IDLGLDLSDSDLD, corresponding to residues 9–21 of HSV-1 ICP27.

### What is the molecular basis of d1-2’s cell-type dependence?

Our characterization of d1-2’s growth in multiple cellular contexts (Fig. 1D) shows that this mutant can replicate to an appreciable extent in four established cell lines: African green monkey-derived Vero and CV-1 cells, and human-derived ARPE-19 and HEK293 cells. In contrast, d1-2 is unable to replicate at all in several human cell lines (HeLa, HEp2, U2OS, U373, HFF-Tert) and two primary human cell cultures (TE286 tonsillar fibroblasts and HFF). Thus, cells appear to fall into two distinct classes with respect to their ability to serve as a host for d1-2. However, inspection of the members of these two classes fails to reveal an obvious cell characteristic, such as species origin, tissue origin, or growth/transformation status, that could be the primary determinant of d1-2 replication. What then is the basis of this cell type-dependence? One hypothesis that we explored is that semipermissive cells are those which are unable to mount an effective type-I interferon response. Supporting this conjecture is the fact that Vero cells are β-interferon gene mutants (49) and that HEK293 cells exhibit deficiencies in type-I interferon expression (60). We asked whether the semipermissive replication of d1-2 in Vero cells could be altered by treating the cells with type-I interferon prior to infection. While this treatment modestly inhibited (~10-fold) d1-2 replication (Fig. 1E), it still allowed far more replication than is seen in restrictive cells. Thus, although we cannot rule out a role for the type-I interferon system, it seems unlikely that its functionality is the central determinant of a cell’s permissivity for d1-2.

Our studies did uncover one potentially important clue for understanding why cells fall into two categories with respect to d1-2 replication. We found that d1-2 and d27-1 are incapable of replicating their DNA in HEp2 cells, but can do so in Vero cells. Thus, it can be concluded that ICP27 carries out a function essential for viral DNA replication in HEp2 cells and that the d1-2 mutant is defective for this function. These results suggest that the central feature of a cell’s permissivity for d1-2 involves the process of HSV-1 genome replication (reviewed in 61 and 62). To pursue this idea further, it would be interesting to survey additional cells used in Fig. 1D to see if the correlation between d1-2 restriction and the requirement for ICP27 in viral DNA synthesis is maintained. If so, experiments could be done to explore ICP27’s role in viral genome replication in more depth. In this regard, it has been shown that ICP27 enhances viral DNA synthesis in Vero cells by upregulating expression of the seven E genes encoding the viral DNA replication factors (63). It would be interesting to investigate whether the expression of these genes is even more dependent on ICP27 in HEp2 and other restrictive cells.

### ICP27 NES mutants are deficient in ICP4 expression.

**FIG 10:**
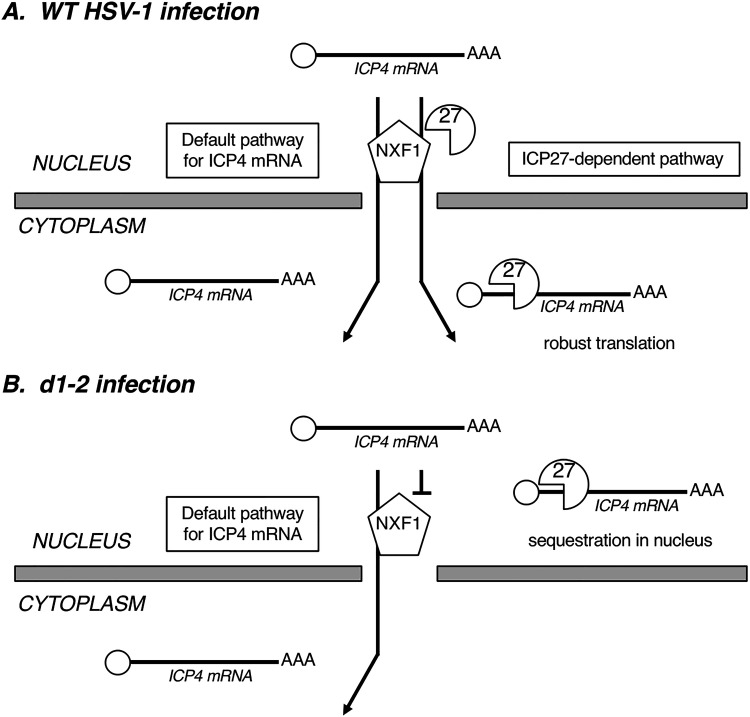
Models for the role of ICP27 in the early expression of ICP4 mRNA in cells infected by WT HSV-1 (A) or d1-2 (B). See text for details.

Perhaps our most unexpected finding in this study is that certain HSV-1 ICP27 mutants are deficient in ICP4 expression at early times postinfection. This suggests a heretofore unappreciated role for ICP27 in the induction of this critical viral transcription factor. Given ICP27’s well-accepted function in mediating the export of intronless viral E and L transcripts, it is interesting to speculate that ICP27 also promotes ICP4 mRNA export. This is consistent with the fact that the ICP4 transcript is one of the two IE transcripts (along with that encoding ICP27 itself) which are unspliced, and therefore, unable to recruit TREX factors via mRNA splicing. Based on this idea, we propose a model to explain how ICP27 enhances ICP4 expression during the WT HSV-1 infection (Fig. 10A). The model posits that following VP16-induced transcription of the ICP4 gene, capped and polyadenylated ICP4 mRNA is exported to the cytoplasm by two distinct mechanisms. The first is a “default” mechanism, likely involving the innate ability of the ICP4 transcript to recruit TREX components independently of splicing, as has been documented for several intronless host cell mRNAs (40, 64–66). Such a mechanism is consistent with the observation that ICP4 can be expressed in the absence of infection in transfected cells (29), as well as in ICP27 null mutant infections (e.g., see Fig. 4 and 5). The second mechanism is an ICP27-dependent one, as occurs for numerous HSV-1 E/L transcripts. A crucial point is that while both pathways would enable mRNA export, the ICP27-dependent pathway offers a potential advantage for ICP4 expression, as ICP27 can also enhance the translation of viral mRNAs to which it is bound (32, 56, 57).

This model also has the potential to explain why accumulation of ICP4 mRNA is reduced in the cytoplasm of d1-2-infected HEp2 cells (Fig. 10B). In this case, ICP27 would still be expected to bind to the GC-rich ICP4 mRNA in the nucleus via its RGG box, but would be unable to access NXF1 for export to the cytoplasm. This would result in sequestration of ICP4 mRNA in the nucleus, leading to lower cytoplasmic levels of this IE transcript, as we have documented (Fig. 9B, panel i). Supporting this, ICP27 has been shown to be tightly confined to the nucleus in d1-2 and dLeu infections, in contrast to its whole-cell distribution in the WT infection (29).

Clearly, additional work is required to understand how ICP27 regulates expression of ICP4 mRNA, and why the results differ between cell lines. However, based on our findings here, we conclude that ICP27 can enhance ICP4 expression in some circumstances, and that altered forms of ICP27 can actively interfere with ICP4 expression. These results suggest the existence of an additional layer of regulation for the ICP4 gene beyond the parallel activation of IE genes by VP16. Given the central importance of ICP4 in driving the productive HSV-1 infection, such a multitiered regulatory scheme would not be unexpected.

## MATERIALS AND METHODS

### Cells, viruses, and infections.

The cell lines Vero, CV-1, 293, U2OS, ARPE19, HeLa, and HEp2 were obtained from American Type Culture Collection. The human glioblastoma cell line U373 was a gift from the late John Ohlfest of the University of Minnesota. V27 cells are Vero cell derivatives that have been stably transfected with the HSV-1 ICP27 gene (15). Human foreskin fibroblasts (HFF) and life-extended HFF-TERT cells (67) were kindly donated by Dr. Wade Bresnahan of the University of Minnesota. Primary human tonsillar fibroblasts (TE286 cells) were derived at the University of Minnesota using palatine tonsil from a 20-year-old female patient and a standard published protocol (68). All cells were grown in Dulbecco-modified Eagle medium containing 5% or 10% heat-inactivated fetal calf serum (FCS), 50 U/mL penicillin, and 50 μg/mL streptomycin.

The WT strain of HSV-1 used in this study was KOS1.1. The ICP27 mutants used were all derivatives of KOS1.1, and consisted of d27-1 and n504R (15); dLeu and dAc (29); d1-2 (44); d3-4 and d4-5 (46); d2-3, d5-6, and d6-7 (29); and M11, M15, and M16 (45). The ICP0 mutant used was n212 (69). Infections were performed in phosphate-buffered saline (PBS) containing 0.1% glucose and 0.1% heat-inactivated newborn calf serum. Viral adsorption was for 1 h at 37°C, after which time the viral inoculum was replaced with 199 medium containing 1% heat-inactivated newborn calf serum, 50 U/mL penicillin, and 50 μg/mL streptomycin. For experiments to assess viral growth, infected cells were subjected to a low pH acid-glycine buffer wash step at 2 hpi, to inactivate residual extracellular virus (70). For the interferon sensitivity experiment shown in Fig. 1E, cells were pretreated before infection with 1,000 U of human lymphoblastoid IFN-α (Sigma) per mL. HSV-1 plaque assays were carried out on Vero or V27 cells as described (47). Plaque assays for the n212 mutant were done on U2OS cells, which complement the growth defects of ICP0 mutants (71).

### ICP27 expression plasmids and d27-1 complementation assays.

The WT ICP27 expression vector pBH27 has been described (72), as has the pBH27ΔLeu plasmid that carries the dLeu mutation (29). Additional expression plasmids having the N-terminal ICP27 mutations shown in Fig. 2A were engineered using the following strategy. First, plasmids containing the desired mutations in a 374 bp AgeI-DraIII fragment of the ICP27 gene were purchased from Genescript. The mutated sequences were then introduced into the intact ICP27 gene by digesting ICP27 expression plasmid pBS27 (44) with AgeI and DraIII, and replacing the WT AgeI-DraIII fragment with the mutant fragment. All mutations were confirmed by DNA sequencing.

The d27-1 complementation assay has been described (44, 53). Briefly, subconfluent 25 cm^2^ (or in some cases 12.5 cm^2^) flasks of HEp2 or Vero cells were transfected with 7.5 μg (or 3.25 μg in the case of 12.5 cm^2^ flasks) of ICP27-encoding plasmid using Lipofectamine 2000 (Invitrogen). At 24 h posttransfection, cells were infected at an MOI of 1 PFU/cell with d27-1. At 2 hpi, flasks were subjected to a low pH wash step to inactivate extracellular virus, as described above. Infections were terminated at 24 hpi by adding a volume of sterile nonfat dry milk that was equal to the media volume and freezing at −70°C. Virus was released by three cycles of freeze-thawing, and virus yields were determined by plaque assay of the lysates on V27 cells. All experiments included pBH27 as the positive-control plasmid and cloning vector pUC19 as the negative control. Each experiment was done in biological triplicate, and each plasmid was analyzed in two to four independent experiments. To determine the relative complementation efficiency, the ratio of complementation in Vero cells to that in HEp2 cells was determined for each experiment. These values were used to determine the mean relative complementation efficiencies shown in Fig. 2C.

The d1-2 complementation experiment shown in Fig. 8 was performed using the protocol described above. pBH27 was the ICP27 expression plasmid used. Plasmid pK1-2, a gift of Neal DeLuca, was used for ICP4 expression (73).

### Analysis of HSV-1 DNA replication.

Triplicate cultures of Vero or HEp2 cells in 25 cm^2^ flasks were mock-infected or infected with WT HSV-1 or ICP27 mutant (d1-2 or d27-1) at an MOI of 10 PFU/cell. At 2 and 20 hpi, infected cells were harvested in PBS by scraping and low-speed centrifugation. Total DNA was isolated from the resuspended cells using QIAamp DNA Mini Kits (Qiagen). HSV-1 DNA levels, relative to a cellular gene, were then determined by qPCR. For standardization, cell DNA standards were generated by making 10-fold serial dilutions of mock-infected Vero cell DNA, starting at 100 ng/μL. HSV-1 DNA standards were generated by making 10-fold serial dilutions of plasmid pcDNA22 (carrying the HSV-1 ICP22 gene) (74) starting at 100 ng/μL. qPCRs were performed in duplicate with 25 μL reaction mixtures in a Bio-Rad iQ5 real-time detection system. Each reaction contained 5 μL SsoAdvanced Universal SYBER Green Supermix (Bio-Rad), 2 μL PCR-grade water, 2.5 μL template DNA, and either 0.25 μL of 1 μM (1.5 μg) primers for the ICP22 gene (FW: 5′-TTT GGG GAG TTT GAC TGG AC-3′/RV: 5′-CAG ACA CTT GCG GTC TTC TG-3′) (75) or 0.25 μL of 1 μM (1.5 μg) primers for the cellular gene 1,3-alpha-galactosyltransferase gene (FW: 5′-ACA CCC TAG GCC AGT CAG TG-3′/RV: 5′-TGC ATG CTG ACT CTT TC-3′) (76). Reactions were carried out using an annealing temperature of 60°C. Melt curve analysis confirmed the specificity of each PCR. Each primer set amplified their target gene with 95% to 105% efficiency, as calculated by a linear regression performed on a plot of threshold cycle value (Cq) versus log quantity DNA from each standard curve (R^2^ 0.90 to 0.98). Data were analyzed using the Pfaffl method (77). HSV-1 DNA levels were normalized for differences in cell recovery using the cellular gene signal. For each experimental analysis, Vero and HEp2 samples were analyzed in separate qPCR runs. For each analysis, the level of WT HSV-1 DNA at 2 hpi was assigned the value of 1.0.

### Immunoblotting analysis.

Immunoblotting was performed as described previously (45, 78). The following antibodies were used to detect viral proteins: ICP27, mouse monoclonal antibody (MAb) H1113 (Virusys) or H1119 (Rumbaugh-Goodwin Institute); ICP4, mouse MAb H1114 (Rumbaugh-Goodwin Institute for Cancer Research); ICP0, mouse MAb H1112 (Rumbaugh-Goodwin Institute for Cancer Research, ICP22, rabbit polyclonal antibody (a gift from John Blaho), ICP8, mouse MAb H1115 (Rumbaugh-Goodwin Institute for Cancer Research), gD, mouse MAb H1103 (Rumbaugh-Goodwin Institute for Cancer Research); gB, mouse MAb HA-056 (Virusys); VP22, rabbit polyclonal antibody (34) (a gift from Gill Elliott); and gC: mouse MAb H1104; Rumbaugh-Goodwin Institute). Host cell proteins ALYREF and β-actin were detected using mouse MAbs 11G5 (EMDMillipore) and AC-15 (Abcam), respectively. For the quantitative analysis shown in Fig. 5, cells were infected in biological triplicate and analyzed by immunoblotting. After blotting, filters were cut horizontally and the top portion was probed for ICP4 while the bottom was probed for ALYREF. Digital images of resulting immunoblots were quantitated using ImageJ software. Levels of ICP4 were normalized for cell recovery using the ALYREF loading controls.

### RNA analysis.

For whole-cell RNA analyses, Vero and HEp2 cells were infected with WT HSV-1 or ICP27 mutants at an MOI of 10 PFU/cell and total RNA was isolated at 3 hpi using TRIzol (Invitrogen). The quality and quantity of RNA were determined by Qubit RNA assay (Thermo Fisher Scientific). Note that purification of the RNA included a DNase I (RNase-free; Sigma) DNA digestion step and that control experiments showed that the primers designated below failed to amplify DNA when DNase I-treated RNA was used as a template without a reverse transcription step. cDNAs were synthesized from 1 μg of isolated RNA using Qscript cDNA synthesis master mix (Quanta Bio) according to the manufacturer’s protocol. Quantitative PCR (qPCR) was performed with a Bio-Rad CFX96 qPCR system using SSO SYBR green Supermix (Bio-Rad). The primers used to detect ICP4 mRNA were ICP4-F: ACT TAA TCA GGT TGT TGC; ICP4-R: GAA GTT GTG GAC TGG GAA GG; and those used for 18S rRNA were 18S rRNA-F: AGG AAT TGA CGG AAG GGC AC; 18S rRNA-R: TTA TCG GAA TTA ACC AGA CA. To correct for differences in cell recovery, the ICP4 RT-qPCR data were normalized using the 18S rRNA data. To do this, the 18S rRNA Ct values were subtracted from the corresponding ICP4 Ct values.

For RNA analyses from cytoplasmic and nuclear fractions, the cells were subjected to biochemical fractionation. Following trypsinization, the infected cells were collected by centrifugation for 5 min and lysed by resuspension in cytoplasmic lysis buffer (20 mM HEPES pH 7.9; 10% glycerol; 100 mM NaCl, 1.5 mM MgCl_2_; 0.05% NP40 with protease and RNase inhibitors) and incubated for 5 to 10 min on ice before centrifugation for 5 min at 4000 rpm at 4°C. Supernatants were collected and saved as the cytoplasmic fractions. The nuclear pellets were washed with the same buffer once following centrifugation and saved as the nuclear fractions. RNA from these fractions was isolated by TRIzol (Invitrogen) and reverse transcription quantitative PCR (RT-qPCR) was carried out as described above. The ICP4 mRNA and 18S rRNA primers used are listed above; the ICP0 mRNA primers used were ICP0-F: AAG CTT GGA TCC GAG CCC CGC CC; ICP0-R: AAG CGG TGC ATG CAC GGG AAG GT. Normalization of ICP4 and ICP0 levels to those of 18S rRNA was done as described above. Note that 18S rRNA, or its precursor forms, are found in both cytoplasmic and nuclear compartments (79). Successful fractionation was confirmed by the detection of the highly nuclear-restricted BORG lncRNA (80) in the nuclear but not cytoplasmic fractions using the following primers: hBORG-F: CGA TGG GT CAC ATG ACA AAG; hBORG-R: TTC TCC TGG GGG AAA AAT AGA.
